# Control of social monogamy through aggression in a hermaphroditic shrimp

**DOI:** 10.1186/1742-9994-8-30

**Published:** 2011-11-11

**Authors:** Janine WY Wong, Nico K Michiels

**Affiliations:** 1 Animal Evolutionary Ecology, Department for Biology, University of Tübingen, Auf der Morgenstelle 28, D-72076 Tübingen, Germany; 2 Zoological Institute, Evolutionary Biology, University of Basel, Vesalgasse 1, CH-4051 Basel, Switzerland

**Keywords:** hermaphrodite, aggression, elimination, moult, monogamy, pair stability, group size, food competition

## Abstract

**Introduction:**

Sex allocation theory predicts that in small mating groups simultaneous hermaphroditism is the optimal form of gender expression. Under these conditions, male allocation is predicted to be very low and overall per-capita reproductive output maximal. This is particularly true for individuals that live in pairs, but monogamy is highly susceptible to cheating by both partners. However, certain conditions favour social monogamy in hermaphrodites. This study addresses the influence of group size on group stability and moulting cycles in singles, pairs, triplets and quartets of the socially monogamous shrimp *Lysmata amboinensis*, a protandric simultaneous hermaphrodite.

**Results:**

The effect of group size was very strong: Exactly one individual in each triplet and exactly two individuals in each quartet were killed in aggressive interactions, resulting in group sizes of two individuals. All killed individuals had just moulted. No mortality occurred in single and pair treatments. The number of moults in the surviving shrimp increased significantly after changing from triplets and quartets to pairs.

**Conclusion:**

Social monogamy in *L. amboinensis *is reinforced by aggressive expulsion of supernumerous individuals. We suggest that the high risk of mortality in triplets and quartets results in suppression of moulting in groups larger than two individuals and that the feeding ecology of *L. amboinensis *favours social monogamy.

## Introduction

Sex allocation theory predicts optimal investment into male and female function in sexually reproducing organisms [1] and has been the focus of many studies (for example [2-6]). In separate sex species, sex allocation determines the sex ratio in a population whereas in hermaphrodites allocation is optimized within an individual [7]. Here, optimal sex allocation is the value that maximizes the product of the fitness derived from both male and female investment [1]. Assuming a direct trade-off between resources invested in the male and female function, any amount of resources devoted to sperm production implies a reduction in resources available for the production of eggs and vice versa. One key factor that influences sex allocation in simultaneous hermaphrodites is mating group size [1], i.e. the number of actual mating partners. A small mating group will induce competition among self sperm of a sperm donor (called local sperm competition or LSC by Schärer [6]) and favour smaller ejaculates, leading to reduced allocation to the male function. With increasing group size, the amount of reproductive competition through the male function increases, which results in higher optimal allocation to sperm production in large groups [1,8-12]. A direct consequence is that hermaphrodites that live in pairs benefit from the absence of sperm competition between multiple partners. Here, sperm donors only need to produce just enough sperm to fertilize their partner, while maximizing the amount of resources available for eggs. In the end, this will result in the highest possible per capita reproductive success. Furthermore, the maintenance of monogamy assures the permanent presence of a mating partner [13] and increases defensive success of a specific microhabitat (e.g. host or refuge) through reciprocity or mutualism [14]. However, monogamy is highly susceptible to cheating. Extra-pair matings in the male role offer an increase in reproductive success, because sperm are cheaper to produce than eggs [15].

Nevertheless, social monogamy might be favoured under certain conditions independent from sex allocation theory. Baeza [16] showed that high predation risk, host scarcity and small host size lead to social monogamy and female-biased sex allocation in the marine shrimp *Lysmata pederseni*. *Lysmata *shrimp are protandric simultaneous hermaphrodites [17-19]. They all mature as functional males and later on attain the female sexual function. Individuals have thus the ability to reproduce as both male and female [18,20,21]. However, the sociobiology of *Lysmata *shrimp is highly variable. Some species live in large groups (aggregations), whereas others live in small groups or even in pairs (social monogamy) [17,22]. The pair living species (*L. amboinensis, L. grabhami, L. debelius*) can be grouped in the 'cleaner' clade, due to their specialized fish-cleaning behaviour [17,22]. What are the conditions that favour social monogamy in these species? We suggest that it is their feeding ecology. Cleaner shrimp are territorial and highly dependent upon their clients for food [23]. Chapuis & Bshary [24] showed that clients occasionally jolted during cleaning interactions with the cleaner shrimp *Periclimenes longicarpus*. Those jolts are a correlate of cheating the client (i.e. eating the client's mucus instead of ectoparasites) in the cleaner wrasse (*Labroides dimidiatus*) and are usually followed by aggressive chasing or abandonment of the cleaner [25]. Chapuis & Bshary [24] found a positive correlation between the jolt frequency and the maximum number of shrimp cleaning. Consequently, the risk of loosing fish clients due to cheating cleaners rises with enlarged group size. A high number of shrimp may increase competition for the cleaning station and access to clients. By keeping the number of individuals in a group low, which would otherwise have to share clients, the potential for conflicts and food competition are minimized.

In this study, we investigated how group size affects moulting cycle and group stability in *L. amboinensis*. Individuals were set up in singles, pairs, triplets and quartets. With increasing group size, the number of potential mating partners rises as well as the number of competitors. If *L. amboinensis *maintains a fixed group size of two, we expected to see more aggression in groups larger than pairs. Furthermore, as stress can delay moulting in crustaceans [26-29], we also expected to see fewer moults in triplets compared to pairs. Depending on whether quartets would split in two stable pairs or not, we expected them to either be similar to pairs or triplets regarding aggression and moulting. The single treatment provided a baseline for moulting cycles and mortality.

## Results

### Mortality in triplet and quartet treatments

**Figure 1:**
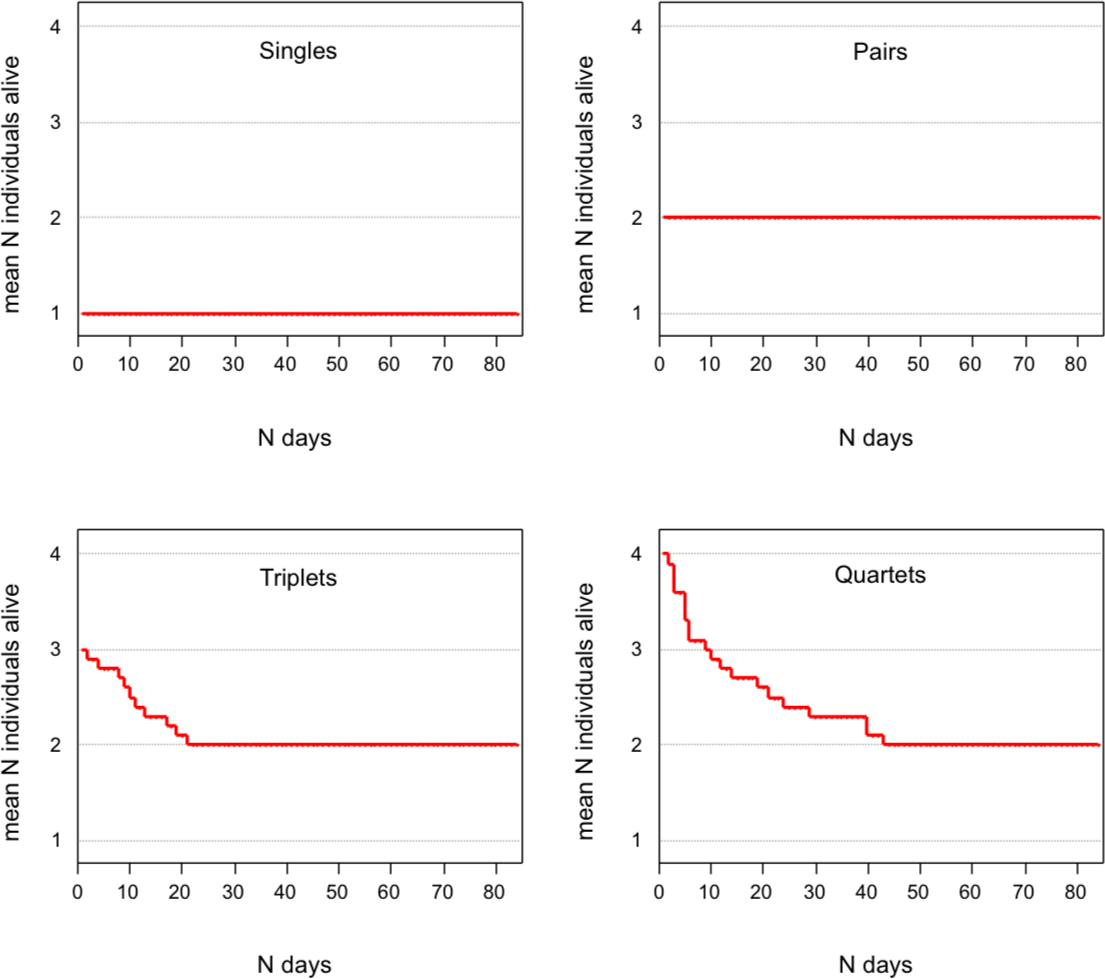
**Mean number of *L. amboinensis *individuals alive across time**. The survival diagram shows the mean number of shrimp alive per tank across time for each treatment group (singles, pairs, triplets and quartets).

We observed selective mortality in the triplet and quartet treatments and no mortality in the single and pair treatments. The pattern of mortality was such that, after 42 days, one individual in each triplet and two individuals in each quartet treatment had died (Figure 1). In all cases (N = 30) the individuals died shortly after moulting as leftovers of the exuvia could be found the next morning. Analysis of nighttime-videos indicated that aggressive interactions have contributed to mortality events in the triplet and quartet treatments. Survival was 100% once all triplets and quartets had turned into pairs. All resultant pairs survived the remaining experimental period. The time until the first individual died was not significantly different between triplet and quartet tanks (Log-Rank, *χ^2 ^*= 1.394, *df *= 1, *p *= 0.238), suggesting that the time of mortality was independent of group size.

**Figure 2:**
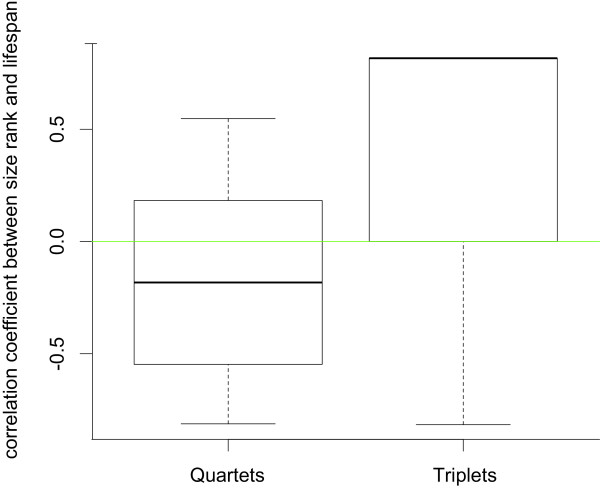
**Comparison of the correlation coefficient between size rank and lifespan**. The boxplots show the range of Kendall's τ correlation coefficient from the correlation between size rank and lifespan for quartets (left) and triplets (right).

We tested the effects of size rank and moulting sequence within each tank on the lifespan of every individual as well as the effect of size rank on moulting sequence. The differences between the groups (triplets, quartets and zero) were not significant for the correlation coefficient between moulting sequence and lifespan (Kruskal-Wallis, *χ^2 ^*= 0.364, *df *= 2, *p *= 0.832) or size rank and moulting sequence (Kruskal-Wallis, *χ^2 ^*= 0.776, *df *= 2, *p *= 0.679). But we found a significant overall difference between the groups for the correlation coefficient between size rank and lifespan (Kruskal-Wallis, *χ^2 ^*= 7.274, *df *= 2, *p *= 0.026). In the subsequent pairwise comparisons between all groups we found that Kendall's τ between size rank and lifespan differed significantly between triplets (median = 0.817) and quartets (median = -0.183) (Steel-Dwass*, Z *= -2.598, *N *= 10, *p *= 0.025) (Figure 2). However, the correlation coefficient between size rank and lifespan was not significantly different from zero in triplets (Steel-Dwass, *Z *= 0.927, *N *= 10, *p *= 0.623) and in quartets (Steel-Dwass, *Z *= -0.484, *N *= 10, *p *= 0.879).

### Number of moults

**Figure 3:**
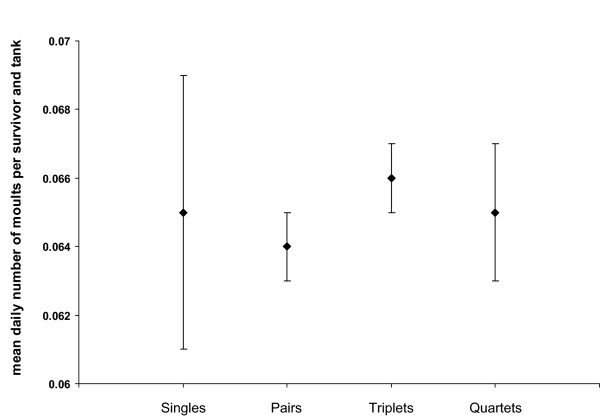
**Comparison of the daily number of moults per survivor and tank**. The black diamonds show the mean daily number of moults per *L. amboinensis *individual and tank for all treatment groups containing one (singles) or two (pairs, triplets, quartets) shrimp. That is after triplet and quartet treatments became pairs due to mortality of conspecifics. Error bars indicate standard errors.

As predicted, we found a significant increase in the number of moults with time, i.e. individuals of triplet and quartet treatments had a higher number of moults once living in pairs (MANOVA, within-subjects effect of time: *F *= 0.303, *df *= 1,18, *p *= 0.031) (daily number of moults per survivor and tank; mean ± SE: before changing to pairs: 0.049 ± 0.007, after changing to pairs: 0.065 ± 0.001). There were no significant effects of the treatment group (MANOVA, *F *= 0.106, *df *= 1,18, *p *= 0.184) or the interaction between treatment and time (MANOVA, *F *= 0.110, *df *= 1,18, *p *= 0.177). When comparing the daily number of moults per survivor and tank between all treatment groups after triplets and quartets had changed to pairs, i.e. when there were two individuals in all groups except for the singles, there was no significant difference in the number of moults between individuals from single, pair and group treatments that had changed to pairs (Welch ANOVA, *F *= 0.184, *df *= 3,14.745, *p *= 0.905) (Figure 3).

## Discussion

We recorded group size regulation and moulting frequency in singles, pairs, triplets and quartets of the pair-living hermaphroditic shrimp *L. amboinensis*. We found selective mortality of shrimp in triplets and quartets, which ceased as soon as a group size of two was achieved. No fatalities occurred in the single or pair treatments. A higher number of moults was found in the survivors of triplet and quartet treatments after changing from groups to pairs.

We explain mortality in triplets and quartets as result of the elimination of competitors, indicating behavioural control over a stable group size of two. Even though shrimp were kept under *ad libitum *food conditions, we assume that the elimination of supernumerous individuals is intrinsic, as individuals will most likely face food limitations in the field. A group size of three would lead to higher food competition and diminished growth. Since body size is correlated with egg number in shrimp [16,30], the diminished growth rate would lead to a decrease in fecundity in all individuals of the group. The same would apply for group sizes larger than three individuals. In the long run it would be more profitable to eliminate permanent food competitors and have a single, more fecund mating partner in a group size of two (see also [16]). Perceiving other conspecifics as direct competition rather than potential mates was reported for the simultaneous hermaphroditic worm *Ophryotrocha diadema *[3]. A higher number of intolerant acts in groups compared to pairs was observed when testing for changes in sex allocation. In contrast to our findings, other studies showed that mating group size can be high in simultaneous hermaphrodites and that social group size can be positively linked to the number of mating partners [31,32].

Furthermore, we found that mortality in shrimp occurred during the night in individuals that had always just moulted. During daytime no aggressive acts towards shrimp that died later in the course of the experiment were observable (personal observation). Shrimp are most vulnerable when just moulted, which happens at night. This may be the only point in time when an individual of approximately the same body size is able to harm or even kill a conspecific. It may explain why mortality occurred in freshly moulted individuals only and why aggression was absent during daytime. We neither found any significant overall effects of size rank and moulting sequence on lifespan nor any effect of size rank on moulting sequence. However, the effect of size rank on lifespan was significantly different between triplets and quartets, suggesting that the pattern of mortality was dissimilar in groups of three compared to four individuals. The smallest individuals died more frequently in triplets compared to quartets. As the ratio of aggressors to moulted individuals was higher in quartets compared to triplets, larger individuals could have become victims of aggressive interactions more often in groups of four compared to three individuals. An obvious pattern of mortality sequence in quartets remains obscure though.

We predicted to find fewer moults in groups larger than two individuals due to stress. According to this prediction, our results showed a lower number of moults in groups before changing to pairs. We suggest that the high risk of mortality after moulting in triplets and quartets results in suppression of moulting in groups compared to pairs and singles. After the elimination of competitors in triplets and quartets the number of moults increased to the same level as in singles and pairs. We propose that the higher variation in the number of moults in singles (see Figure 3) results from isolation of the respective individuals. The presence of a mating partner might trigger ovarian development, moulting and spawning. Even though mating partners were absent, we occasionally observed spawning of unfertilized eggs in a few singles, but not in others. This might have lead to differences in the length of the intermoult intervals and thus to the higher variation in the number of moults. Fiedler [21] also reported that singles of *L. amboinensis *drifted from a regular moulting cycle.

Given the fact that social monogamy can only be found in *Lysmata *species that adopted cleaning behaviour and a symbiotic lifestyle [22], we propose that the feeding behaviour of *L. amboinensis *inhibits group sizes larger than two individuals. Additional shrimp possibly peril the established cooperation between the shrimp and their clients, which would lead to an increase in food competition and thus a reduction in individual fecundity. Other studies [33,34] found that the overall proportion of cooperating group members decreased significantly with increasing group size. They also emphasized the greater opportunity for cheating in larger groups. In our study, the feeding ecology of *L. amboinensis *combined with restricted space available in tanks to occupy distinct 'cleaning stations' might consequently explain why quartets did not form two stable pairs. However, we do not expect to see the same extreme level of aggression due to increased group size in the field. Here, individuals should prevent perilous conflicts by avoiding close proximity to established pairs on a cleaning station. By experimentally forcing individuals to stay together in a small area, we discovered a causal explanation for how a simultaneous hermaphrodite can maintain social monogamy. This mechanism would have been overlooked in a field study.

## Conclusion

In conclusion, our results show that *L. amboinensis *adjusts its group size by eliminating supernumerous individuals until establishing monogamous pairs. Aggressive group size diminution could be a mechanism to limit competition and to ensure cooperation between partners when providing cleaning services. The number of moults increased after the adjustment of triplets and quartets to pairs. We interpret the lower number of moults as a result of the high risk of mortality when living in groups larger than two individuals. Future experiments should test the effects of group size enlargement in *L. amboinensis *in the field. Moreover, the causes and mechanisms of the maintenance of monogamy should be explored in other pair-living species of *Lysmata *shrimp, which share the same feeding ecology. These findings could be related to other, non-monogamous hermaphrodites and theoretical models.

## Materials and methods

### Model species

*Lysmata *shrimp are protandric simultaneous hermaphrodites. An individual matures as a functional male and later on, with increasing body size, gains the female function to become a fully functional simultaneous hermaphrodite [18,21]. *L. amboinensis *De Man (Caridea: Hippolytidae) was classified as 'pairs' species by Bauer [18] and as 'tropical-pairs' species by Baeza [35] due to its social monogamy. It is small in size (approx. 6 cm total length) [21] and occurs in the tropical waters of the Indo-Pacific or the Red Sea. It is often referred to as cleaner shrimp due to the cleaning behaviour it performs on fish clients [17,22].

As in all caridean species, individuals mating as females undergo a moult prior to mating and spawning [36]. Only for a few hours after moulting, hermaphrodites are receptive and can receive sperm from a male phase or another hermaphroditic phase partner [37]. Thus, the sex roles are defined by the moulting cycle of each individual. The mean intermoult interval reported from Fiedler [21] ranged from 20.8 to 18.3 days, but was much shorter in our experiment (approx. 15 days in pairs). Fertilization is external [36]. The male role can be attained anytime, even while incubating eggs. A mating event is generally non-reciprocal [18,38,39]. Although it is mechanically possible self-fertilization does not occur [21,39]. There is no long-term sperm storage [14,38,39].

### Maintenance of shrimp

*L. amboinensis *was obtained from De Jong Marinelife B.V. (Lingewaal, Netherlands) and imported from the Philippines. Shrimp were allowed to acclimatize to laboratory conditions for 31 days. We did not have precise information about the age of each shrimp, but individuals should not have differed in their behaviour as we assured by the presence of eggs that the hermaphroditic phase was accomplished in all individuals. The single treatment was a control to assign the frequency of moulting in the absence of a mating partner. Subsequently, shrimp were separated into treatment groups in a size-controlled randomized manner. Carapace length (CL) was used to assort candidates of approximately the same body size into the same tank to minimize size effects. The different body size classes were spread evenly among treatment groups. The remaining size differences between the largest and the smallest individuals within one tank were (mean ± SE) 0.038 mm ± 0.010 mm in pairs, 0.148 mm ± 0.065 mm in triplets and 0.211 mm ± 0.047 mm in quartets.

Individuals were allocated to one of four treatments: single shrimp were kept in 15 l (25*25*25 cm), pairs in 30 l (35*35*25 cm), triplets in 45 l (42*42*25 cm) and quartets in 60 l (49*49*25 cm) aquaria, with the same water level (height of tanks = 25 cm), resulting in an equal surface area per individual. All tanks were connected to a single water circulation system (1440 l). The ground of each aquarium was covered with coral sand. Shrimp were maintained at 24 to 25 °C water temperature, 34 to 35 ‰ salinity (Tropic Marin^® ^Sea Salt) and a 12 h light:12 h dark cycle. Partial water change (200 l) in the circulatory system and removal of debris were carried out weekly.

Each treatment consisted of 10 replicates (pairs: N = 20 shrimp, triplets: N = 30 shrimp, quartets: N = 40 shrimp) except for the single treatment, which consisted of 6 aquaria (N = 6 shrimp). Aquaria were spatially randomized across a three-level rack. Opaque PVC plates between tanks prevented visual contact. To minimize visual stress within tanks they were compartmentalized into zones with incomplete opaque dividers. The number of zones was equal to the number of shrimp in the tank. Individuals were allowed to move freely throughout the aquarium. Every zone contained one plastic perch with a uniform overhang. Personal observations of *L. amboinensis *have shown that individuals prefer to sit on some structures (rocks, corals or artificial perches) rather than simply on the ground. Offering perches increased the likelihood that shrimp show their natural behaviour. Shrimp were fed daily (Tropical^® ^Shrimp Sticks), resulting in *ad libitum *food condition.

### Measurements

The size of all shrimp was measured before the experiment started. We used carapace length (CL), defined as the distance from the posterior-most margin of the eye orbit to the mid-dorsal posterior margin of the carapace [40], measured to the nearest of 0.01 mm. We used digital photographs (Olympus CAMEDIA C-8080), which were taken in a standardized way and measured CL using the image analysis software ImageJ 1.39e.

Shrimp were marked individually within each tank by clipping short parts of the distinct antennae to record moulting events. Antennae regenerated after every moult and were re-clipped in the same way. We could not detect signs of stress or harm caused by this treatment. Clipping allows indentifying exuviae from moulted individuals, which is not true for coloured elastomer tags, which are injected into the abdominal musculature [39]. Each individual was checked daily, noting each moult the morning after. Exuviae were removed from the aquaria. The date and shrimp ID were noted in case of mortality.

Video observations of newly assembled shrimp quartets were carried out after the experiment in order to reveal the reason for mortality in groups. We used quartet tanks and groups of 4 individuals accordingly (N = 6 replicates). Recordings were done at night using infrared illumination (two infrared spotlights: LED - Infrared Illumination, Model: IR880/12), five cameras (35x Zoom Day&Night IR Cut Auto Iris Vari Focal Camera, Model: CCD1000H35/3.6-126) and a digital video recorder (8 Channel Triplex Digital Recorder, Model: DVR008TPX/SA800NC). Observation and data collection took place for 84 days starting September 24, 2007.

### Statistical analyses

To test whether mortality is dependent on the number of conspecifics in the tank, i.e. whether mortality of the first individual occurred sooner in groups of four compared to three individuals, we compared the lifespan of the first individual that died in triplet and quartet tanks using a survival analysis. We did not compare survival between all individuals and all treatment groups, as the data points within one tank were not independent of each other.

Although shrimp within the same tank were chosen to be of approximately same body size, the remaining small size differences may explain mortality. Alternatively, moulting sequence might have determined mortality within each tank. To evaluate mortality in groups, the relationships between body size, moulting sequence and mortality were quantified for triplets and quartets by computing partial Kendall's τ correlation coefficients between lifespan, size rank and moulting sequence within tanks. We calculated the coefficients within tanks first, to avoid pseudoreplication, as the individuals in one tank were not independent of each other. The largest individual in each tank obtained the highest rank regarding size (= 4). The individual that moulted first in each tank obtained the lowest rank regarding moulting sequence (= 1). We used a Kruskal-Wallis-Test to see, if the correlation coefficients (Kendall's τ) of triplets and quartets were significantly different from each other. At the same time we tested if Kendall's τ was significantly different from zero, i.e. we compared three groups in the Kruskal-Wallis-Test: triplets, quartets and zero. If the overall differences were statistically significant, a post-hoc analysis was performed using the Steel-Dwass test for multiple comparisons. We applied non-parametric tests since the residuals of the correlation coefficients were not normally distributed.

To test whether group size had an effect on moulting, we compared the daily number of moults per survivor and tank for triplets and quartets when they were in groups (more than two individuals) or in pairs (only the two survivors) after mortality of conspecifics within the tank. We used a multivariate analysis of variance (MANOVA) for repeated measurements with the daily number of moults per survivor and tank of groups and of the resulting pairs as dependent variables, the group size as between subjects factor and time as within subjects factor including the interaction between treatment and time. Subsequently, we used a Welch ANOVA to compare the daily number of moults per survivor and tank among all treatments after the group treatments had changed to pairs to test whether the remaining pairs originating from triplets and quartets showed a difference in the number of moults compared to singles and pair treatments where no mortality had occurred. All p-values stated are two-tailed. All statistical analyses were carried out using JMP^® ^Version 9.0.2 ^© ^2010 SAS Institute, Inc.

## Competing interests

The authors declare that they have no competing interests.

## Authors' contributions

JWYW conducted the assembly of the tank system, carried out the study, participated in its design and analysis, and drafted the manuscript. NM contributed to the study design, the data analysis and manuscript preparation. All authors read and approved the final manuscript.

## References

[B1] Charnov EL The theory of sex allocation 1982 Princeton: Princeton University Press 7144766

[B2] Angeloni L Bradbury JW Charnov EL Body size and sex allocation in simultaneously hermaphroditic animals Behavioral Ecology 2002 13 419 426 10.1093/beheco/13.3.419

[B3] Lorenzi MC Schleicherova D Sella G Life history and sex allocation in the simultaneously hermaphroditic polychaete worm *Ophryotrocha diadema*: the role of sperm competition Integrative and Comparative Biology 2006 46 381 389 10.1093/icb/icj042 21672750

[B4] Schwanz LE Janzen FJ Proulx SR Sex allocation based on relative and absolute condition Evolution 2010 64 1331 1345 20002168 10.1111/j.1558-5646.2009.00916.x

[B5] Wapstra E Warner DA Sex Allocation and Sex Determination in Squamate Reptiles Sexual Development 2010 4 110 118 10.1159/000272459 20051672

[B6] Schärer L Tests of sex allocation in theory in simultaneously hermaphroditic animals Evolution 2009 63 1377 1405 10.1111/j.1558-5646.2009.00669.x 19245397

[B7] Michiels NK Mating conflicts and sperm competition in simultaneous hermaphrodites Sperm Competition and Sexual Selection 1998 219 254

[B8] Charnov EL Sex allocation and local mate competition in barnacles Marine Biology Letters 1980 1 269 272

[B9] Fischer EA Sexual selection in a simultaneously hermaphroditic coral-reef fish American Naturalist 1981 117 64 82 10.1086/283686

[B10] Fischer EA Local Mate Competition and Sex Allocation in Simultaneous Hermaphrodites The American Naturalist 1984 124 590 596 10.1086/284298

[B11] Lorenzi MC Sella G Schleicherova D Ramella L Outcrossing hermaphroditic polychaete worms adjust their sex allocation to social conditions Journal of Evolutionary Biology 2005 18 1341 1347 10.1111/j.1420-9101.2005.00916.x 16135129

[B12] Schärer L Ladurner P Phenotypically plastic adjustment of sex allocation in a simultaneous hermaphrodite Proceedings of the Royal Society of London Series B-Biological Sciences 2003 270 935 941 10.1098/rspb.2002.2323 PMC1691333 12803908

[B13] Sogabe A Matsumoto K Yanagisawa Y Mate change reduces the reproductive rate of males in a monogamous pipefish *Corythoichthys haematopterus*: The benefit of long-term pair bonding Ethology 2007 113 764 771 10.1111/j.1439-0310.2007.01370.x

[B14] Correa C Thiel M Mating systems in caridean shrimp (Decapoda: Caridea) and their evolutionary consequences for sexual dimorphism and reproductive biology Revista Chilena de Historia Natural 2003 187 203

[B15] Di Bona V Lorenzi MC Sella G Functional males in pair-mating outcrossing hermaphrodites Biological Journal of the Linnean Society 2010 100 451 456 10.1111/j.1095-8312.2010.01435.x

[B16] Baeza JA The symbiotic lifestyle and its evolutionary consequences: social monogamy and sex allocation in the hermaphroditic shrimp *Lysmata pederseni* Naturwissenschaften 2010 97 729 741 10.1007/s00114-010-0689-4 20552156

[B17] Baeza JA Protandric simultaneous hermaphroditism is a conserved trait in *Lysmata *(Caridea: Lysmatidae): implications for the evolution of hermaphroditism in the genus Smithsonian Contributions to Marine Science 2009 38 95 110

[B18] Bauer RT Simultaneous hermaphroditism in caridean shrimps: a unique and puzzling sexual system in the Decapoda Journal of Crustacean Biology 2000 20 116 128

[B19] Bauer RT Same sexual system but variable sociobiology: evolution of protandric simultaneous hermaphroditism in *Lysmata *shrimps Integrative and Comparative Biology 2006 46 430 438 10.1093/icb/icj036 21672755

[B20] Bauer RT Holt GJ Simultaneous hermaphroditism in the marine shrimp *Lysmata wurdemanni *(Caridea: Hippolytidae): an undescribed sexual system in the decapod Crustacea Marine Biology 1998 132 223 235 10.1007/s002270050388

[B21] Fiedler GC Functional, Simultaneous Hermaphroditism in Female-Phase *Lysmata amboinensis* Pacific Science 1998 52 161 169

[B22] Baeza JA Schubart CD Zillner P Fuentes S Bauer RT Molecular phylogeny of shrimps from the genus *Lysmata *(Caridea: Hippolytidae): the evolutionary origins of protandric simultaneous hermaphroditism and social monogamy Biological Journal of the Linnean Society 2009 96 415 424 10.1111/j.1095-8312.2008.01133.x

[B23] Limbaugh C Pederson H A CF Shrimps that clean fishes Bulletin of Marine Science 1961 11 237 257

[B24] Chapuis L Bshary R Strategic adjustment of service quality to client identity in the cleaner shrimp, *Periclimenes longicarpus* Animal Behaviour 2009 78 455 459 10.1016/j.anbehav.2009.06.001

[B25] Bshary R Grutter AS Asymmetric cheating opportunities and partner control in a cleaner fish mutualism Animal Behaviour 2002 63 547 555 10.1006/anbe.2001.1937

[B26] Anger K Spivak E Luppi T Bas C Ismael D Larval salinity tolerance of the South American salt-marsh crab, *Neohelice (Chasmagnathus) granulata*: physiological constraints to estuarine retention, export and reimmigration Helgoland Marine Research 2008 62 93 102 10.1007/s10152-007-0076-5

[B27] Mu XY LeBlanc GA Developmental toxicity of testosterone in the crustacean *Daphnia magna *involves anti-ecdysteroidal activity General and Comparative Endocrinology 2002 129 127 133 10.1016/S0016-6480(02)00518-X 12441123

[B28] Mu XY LeBlanc GA Environmental antiecdysteroids alter embryo development in the crustacean *Daphnia magna* Journal of Experimental Zoology 2002 292 287 292 10.1002/jez.10020 11857462

[B29] Weis JS Cristini A Rao KR Effects of pollutants on molting and regeneration in crustacea American Zoologist 1992 32 495 500

[B30] Bauer RT Wenner A, Kuris A Analysis of embryo production in a caridean shrimp guild from a tropical seagrass meadow Crustacean Egg Production, Crustacean Issues 1991 7 Rotterdam: Balkema Press

[B31] Janicke T Scharer L Determinants of mating and sperm-transfer success in a simultaneous hermaphrodite Journal of Evolutionary Biology 2009 22 405 415 10.1111/j.1420-9101.2008.01660.x 19196388

[B32] Pongratz N Michiels NK High multiple paternity and low last-male sperm precedence in a hermaphroditic planarian flatworm: consequences for reciprocity patterns Molecular Ecology 2003 12 1425 1433 10.1046/j.1365-294X.2003.01844.x 12755872

[B33] Alencar AI Siqueira JD Yamamoto ME Does group size matter? Cheating and cooperation in Brazilian school children Evolution and Human Behavior 2008 29 42 48 10.1016/j.evolhumbehav.2007.09.001

[B34] Bonanni R Valsecchi P Natoli E Pattern of individual participation and cheating in conflicts between groups of free-ranging dogs Animal Behaviour 2010 79 957 968 10.1016/j.anbehav.2010.01.016

[B35] Baeza JA Protandric simultaneous hermaphroditism in the shrimps *Lysmata bahia *and *Lysmata intermedia* Invertebrate Biology 2008 127 181 188 10.1111/j.1744-7410.2007.00122.x

[B36] Bauer RT Remarkable Shrimps - Adaptations and natural history of the carideans 2004 Norman: University of Oklahoma Press

[B37] Zhang D Rhyne AL Lin J Density-dependent effect on reproductive behaviour of *Lysmata amboinensis *and *L. boggessi *(Decapoda: Caridea: Hippolytidae) Journal of the Marine Biological Association of the UK 2007 87 517 522 10.1017/S0025315407053581

[B38] Baeza JA Male mating opportunities affect sex allocation in a protrandric-simultaneous hermaphroditic shrimp Behavioral Ecology and Sociobiology 2007 61 365 370

[B39] Baeza JA Bauer RT Experimental test of socially mediated sex change in a protandric simultaneous hermaphrodite, the marine shrimp *Lysmata wurdemanni *(Caridea: Hippolytidae) Behavioral Ecology and Sociobiology 2004 55 544 550 10.1007/s00265-003-0744-7

[B40] Baldwin AP Bauer RT Growth, survivorship, life-span, and sex change in the hermaphroditic shrimp *Lysmata wurdemanni *(Decapoda: Caridea: Hippolytidae) Marine Biology 2003 143 157 166 10.1007/s00227-003-1043-6

